# 骨髓巨噬细胞M1/M2亚群失衡对免疫介导的新型再生障碍性贫血模型小鼠发病的影响

**DOI:** 10.3760/cma.j.issn.0253-2727.2021.11.010

**Published:** 2021-11

**Authors:** 慧 穆, 惠 贾, 赠华 林, 红慧 郑, 莉 王, 红 刘

**Affiliations:** 1 南通大学医学院，南通 226001 Medical School, Nantong University, Nantong 226001, China; 2 南通大学附属医院，南通 226001 Affiliated Hospital of Nantong University, Nantong 226001, China

**Keywords:** 贫血，再生障碍性, 模型，动物, 巨噬细胞, 炎症介导素类, Anemia, aplastic, Model, animal, Macrophages, Inflammation mediators

## Abstract

**目的:**

探讨巨噬细胞对新型免疫介导的再生障碍性贫血（AA）模型小鼠发病的影响。

**方法:**

以正常雌性B6D2F1（F1）小鼠作为空白对照（对照组，3只），应用双磷酸盐（Clodronate）脂质体尾静脉注射的方法去除小鼠的巨噬细胞（CLO组，4只），以PBS脂质体处理小鼠为阴性对照（PBS组，4只）。并在注射脂质体后以全身辐照（TBI）联合异基因淋巴细胞输注的方法制备F1 AA小鼠模型，分别记为CLO+AA组和PBS+AA组。骨髓活检病理学检查、外周血细胞计数观察各组小鼠AA的程度；流式细胞术检测小鼠骨髓CD4^+^/CD8^+^ T淋巴细胞亚群和巨噬细胞亚群；ELISA法检测各组小鼠外周血血浆中IFN-γ、TNF-α、G-CSF、GM-CSF、TPO、EPO的水平变化。

**结果:**

与PBS+AA组相比，去除巨噬细胞的CLO+AA组小鼠骨髓脂肪化明显改善。PBS+AA组和CLO+AA组的HGB分别为（91.50±31.63）、（110.65±24.15）g/L，PLT分别为（90.85±121.90）×10^6^/L、（461.13±483.45）×10^6^/L，差异均有统计学意义（*P*值均<0.05）。PBS+AA组和CLO+AA组CD4^+^ T淋巴细胞占骨髓细胞的比例分别为（18.50±10.17）％、（7.58±8.00）％；CD8^+^ T淋巴细胞占骨髓细胞的比例分别为（36.23±6.40）％、（6.67±5.78）％。去除巨噬细胞后，CD4^+^和CD8^+^ T淋巴细胞比值均下降，但CD8^+^ T细胞减少程度更大，差异均有统计学意义（*P*值均<0.05）。和PBS+AA组相比，CLO+AA组小鼠的脾脏和骨髓巨噬细胞比例均明显减少，M1型巨噬细胞减少程度明显高于M2型，差异均有统计学意义（*P*值均<0.05）。PBS+AA组和CLO+AA组外周血中IFN-γ水平分别为（602.37±104.62）、（303.01±87.22）ng/L；TNF-α水平分别为（34.46±1.42）、（23.25±4.21）ng/L；GM-CSF水平分别为（9.32±2.00）、（64.85±12.25）ng/L；G-CSF水平分别为（5 891.78±2 632.39）、（17 784.16±488.36）ng/L；EPO水平分别为（9 667.31±4 501.95）、（2 078.02±897.56）ng/L；TPO的浓度分别为（6.36±2.09）、（11.67±2.86）ng/L，差异均有统计学意义（*P*值均<0.05）。

**结论:**

该实验证实在新型F1 AA小鼠模型中巨噬细胞参与了AA的发病。提前去除巨噬细胞，F1 AA模型小鼠的血象和骨髓损伤程度得以改善。正负造血调控因子的改变和巨噬细胞M1/M2亚群失衡可能是造成AA微环境损伤、最终引起AA发病的重要机制。

## Abstract (en)

**Objective:**

To investigate the role of macrophages (Mø) in the pathogenesis of modified immune-mediated aplastic anemia (AA) mice model.

**Methods:**

Before the establishment of the F1 AA mice model by total-body irradiation combined with allogeneic lymphocyte infusion, the mice of the CLO+AA group were treated with clodronate (CLO) liposomes to remove macrophages, and those of the PBS+AA group were treated with phosphate-buffered saline (PBS) liposomes and used as control. The severity of AA was observed by bone marrow (BM) pathological examination and peripheral blood cell count. Flow cytometry (FCM) was used to detect the CD4^+^/CD8^+^ T lymphocyte subsets in the BM and Mø subsets in the BM and spleen of each group. The levels of IFN-γ, TNF-α, G-CSF, GM-CSF, EPO, and TPO in the peripheral blood were detected using enzyme-linked immunosorbent assay. Finally, the relationships between inflammatory factors and Mø subsets were analyzed.

**Results:**

The BM fatty conversion of mice in the CLO+AA group was significantly alleviated compared with the PBS+AA group. Hemoglobin counts were (91.50±31.63) and (110.65±24.15) g/L, respectively, and the platelet counts were (90.85±121.90)× 10^6^/L and (461.13±483.45)×10^6^/L, respectively. The differences were all statistically significant (all *P*<0.05). After removing macrophages, the proportions of CD4^+^ and CD8^+^ T lymphocytes in BM of mice in the CLO+AA group decreased, but the reduction of CD8^+^ T cells was more significant. The proportions of CD4^+^ T cells and CD8^+^ T cells in BM of the PBS+AA group were (18.5±10.17)％ and (36.23±6.40)％, respectively, and in the CLO+AA group were (7.58±8.00)％ and (6.67±5.78)％, respectively. Similarly, the percentage of macrophages in the spleen and BM in the CLO+AA group was significantly reduced compared with the PBS+AA group, most of which were M1 macrophages (*P*<0.05). The levels of IFN-γ in peripheral blood of the PBS+AA and CLO+AA groups were (602.37±104.62) ng/L and (303.01± 87.22) ng/L, respectively, the levels of TNF-α were (34.46±1.42) ng/L and (23.25±4.21) ng/L, respectively, the levels of GM-CSF were (9.32 ± 2.00) ng/L and (64.85±12.25) ng/L, respectively, the levels of G-CSF were (5 891.78±2 632.39) ng/L and (17 784.16±488.36) ng/L, respectively, the levels of EPO were (9 667.31±4 501.95) ng/L and (2 078.02±897.56) ng/L, respectively, and the levels of TPO were (6.36±2.09) ng/L and (11.67±2.86) ng/L, respectively (all *P*<0.05).

**Conclusion:**

This study confirmed that macrophages were involved in the pathogenesis of AA, and the degree of BM damage in AA mice was improved by removing macrophages in advance. The imbalance of M1/M2 macrophages and the changes of IFN-γ and TNF-α may be important mechanisms that eventually lead to AA.

再生障碍性贫血（AA）是一组由多种原因、通过不同机制引起的骨髓造血功能衰竭性疾病。凋亡增加是AA患者全血细胞减少的一个重要原因，而异常激活的T淋巴细胞及其过量分泌的细胞因子IFN-γ和TNF-α则是导致造血细胞过度凋亡的重要因素[1]–[2]。目前AA的一线治疗为免疫抑制治疗（IST）及造血干细胞移植（HSCT），而标准的IST有效率仅50％～70％，提示T细胞免疫攻击以外的因素也参与了AA的发病[2]。

目前的研究已证实，AA患者不仅表现为造血干细胞数量的减少及质量的异常，骨髓微环境也存在异常。骨髓造血微环境主要指非造血细胞来源的龛细胞[3]，巨噬细胞是其重要的组成部分[4]。作为一种具有可塑性和多能性的细胞群体，巨噬细胞在体内外不同环境的影响下，表现出明显的功能差异，具有维持造血平衡、组织再生及炎症反应等多项功能[5]。但有关巨噬细胞与AA的关系，最近才有零星报道。Sun等[6]研究显示，去除宿主的巨噬细胞可以减少骨髓衰竭的发生，首次证实了巨噬细胞与骨髓衰竭的关系。但这项研究中，具体的巨噬细胞亚型变化并未涉及，且国外的AA小鼠模型国内难以复制。因此，本研究中我们应用本课题组改良研制的适合我国条件的F1 AA模型小鼠，验证巨噬细胞与骨髓衰竭的关系，并在此基础上进一步观察外周血中造血调控因子水平的波动及巨噬细胞M1/M2亚型在AA小鼠骨髓及脾脏中的变化，以期进一步了解AA的发病机制。

## 对象与方法

1. 研究对象：B6D2F1（F1）雌性小鼠21只，C57BL/6（B6）雌性小鼠6只，均为清洁级，8～12周龄，体重18～22 g，由南通大学医学院实验动物中心提供。采用随机数字表法将F1小鼠随机分为对照组（3只）、PBS组（4只）、CLO组（4只）、PBS+AA组（5只）和CLO+AA组（5只）。

2. 试剂和仪器：双磷酸盐（Clodronate）/PBS脂质体（荷兰LIPOSOMA公司）；IMDM培养基、RPMI 1640培养基（美国Hyclone公司）；小鼠淋巴细胞分离液（天津灏洋生物制品科技有限公司）；抗小鼠CD3-PE、CD4-PerCP-Cy5.5、CD8-APC，小鼠TNF-α、IFN-γ、G-CSF、GM-CSF、EPO、TPO ELISA检测试剂盒［联科生物技术（杭州）有限公司］；抗小鼠F4/80-Percp-Cy5.5（美国eBioscience公司）；抗小鼠CD11b-FITC、CD86-PE、CD206-Alexa647、CD16/32抗体，Pharmingen固定/破膜试剂盒，流式细胞仪（美国BD公司）；CLINAC-TRILOGY直线加速器（美国VARIAN公司）。

3. 去除小鼠巨噬细胞：造模前1周，CLO组和CLO+AA组的小鼠隔日给予1次200 µl Clodronate脂质体尾静脉注射，共4次；PBS组和PBS+AA组的小鼠在同一时间点尾静脉注射200 µl PBS脂质体。

4. 新型免疫介导的F1 AA模型小鼠的建立：取B6雌性小鼠（与F1同周龄）断颈处死，无菌采集颈部及腋下、腹股沟处的淋巴结，研磨过滤后用IMDM培养基制备淋巴结单个核细胞悬液，调整细胞密度为（1.9～2.1）×10^7^/ml，放置冰上备用。参照文献[7]方法，F1雌性小鼠经直线加速器的辐照设备（剂量为5 Gy）全身均匀辐射后，4～6 h内由腹腔注射0.4 ml B6雌性小鼠淋巴结单个核细胞悬液，保证注射细胞量为（7.6～8.4）×10^6^个。注射完成后的F1小鼠放回原处饲养，注射当天为第0天，饲养12 d F1 AA小鼠模型制备完成。

5. 标本采集：在4次脂质体注射完成后的第2天进行对照组、CLO组、PBS组的标本采集；在小鼠造模后第12天进行CLO+AA组和PBS+AA组的标本采集。摘眼球法取小鼠眶后静脉血1.0～2.0 ml，EDTA抗凝。处死小鼠后，无菌条件下摘取脾脏和左右股骨。脾脏置于200目尼龙滤网研磨制备成脾细胞悬液，再以小鼠淋巴细胞分离液分离获得单个核细胞。

将右侧股骨置于含2 ml RPMI 1640的无菌培养皿中，切除股骨两端，用1 ml注射器抽取RPMI 1640培养基快速冲出骨髓内有核细胞，冲洗4～5次，予90 µm尼龙滤网过滤后转移到15 ml的无菌试管中，洗涤后裂解红细胞，漂洗2次后用1 ml RPMI 1640重悬，再次通过70 µm Falcon细胞滤网过滤，并制成股骨骨髓单个核细胞悬液，以上所有操作均在冰上进行。

无菌分离的小鼠左侧股骨，Bouin固定液固定60 min，乙醇脱水，置于含2 ml Hemapun865甲液的聚乙烯模具中浸泡2 h，将1 ml乙液滴入模具，静置20 min；将包埋好的活检组织切至厚度为4 µm的切片，放在载玻片上置于甲醇3 min后，在Jenner工作液中染色6 min，再放置吉姆萨工作液1 h，蒸馏水冲洗后，分别在醋酸水、95％乙醇中浸渍5～6次，乙醇快速脱水，二甲苯透明后晾干，中性树胶封固，通过光学显微镜进行观察并拍照分析。

6. 外周血血常规检测：采集的小鼠眶后静脉血置于血液混匀器上，按标准操作流程将标本逐个测定分析。

7. 外周血血浆中TNF-α、IFN-γ、G-CSF、GM-CSF、EPO、TPO的水平检测：取ELISA试剂盒中标准品制作标准曲线，按照说明书方法测定小鼠外周血血浆吸光度值，计算各组小鼠外周血血浆中TNF-α、IFN-γ、G-CSF、GM-CSF、EPO、TPO的水平。

8. 骨髓CD4^+^/CD8^+^ T淋巴细胞的检测：取100 µl制备好的骨髓单个核细胞悬液（1×10^7^/ml，单只小鼠）于流式管中，每管分别加入5 µl CD3（PE）、CD4（PerCP-Cy5.5）和CD8（APC）抗体，振荡混匀于4 °C避光孵育30 min；每管加入1 ml流式染色缓冲液，300×*g*离心10 min，弃上清，加入500 µl流式染色缓冲液，上机检测。

9. 骨髓和脾脏巨噬细胞亚型的检测：取100 µl制备好的脾脏或骨髓单个核细胞悬液（1×10^7^/ml，单只小鼠）于流式管中，每管加入2 µl CD16/32抗体进行阻断后，先进行细胞表面染色，每管分别加入1 µg F4/80（Percp-Cy5.5）、CD11b（FITC）和CD86（PE）抗体，振荡混匀于 4 °C避光孵育30 min；PBS清洗1次后，按照Pharmingen固定/破膜试剂盒说明书方法破膜，再加入1 µg CD206（Alexa647）进行胞内染色，孵育45 min，洗涤重悬后上流式细胞仪检测。

10. 统计学处理：采用SPSS 21.0软件进行统计学分析，数据均符合正态分布，以均数±标准差表示，方差齐的资料组间比较采用单因素方差分析，并采用LSD-*t*检验进一步行两两比较；方差不齐的资料组间比较采用Welch检验，并采用Tamhane's 法进一步两两比较，*P*<0.05为差异有统计学意义。

## 结果

1. 小鼠外周血血常规及骨髓组织病理学观察：与正常小鼠比较，PBS+AA组小鼠的HGB、WBC、PLT及ANC均显著下降（*P*值均<0.05）；和PBS+AA组相比，CLO+AA组小鼠的HGB和PLT均显著升高（*P*值均<0.05），和正常对照组相比差异均无统计学意义。PBS+AA组和CLO+AA组的WBC和ANC均明显低于正常对照组（*P*值均<0.05）（表1）。骨髓病理显示，与PBS+AA组相比，CLO+AA组小鼠的骨髓脂肪化程度较轻微，油滴更少（图1）。

**表1:** 各处理组小鼠外周血血常规比较（*x±s*）

组别	鼠数	HGB（g/L）	WBC（×10^6^/L）	PLT（×10^6^/L）	ANC（×10^6^/L）
对照组	3	132.5±27.2	6.03±2.08	624.00±375.95	4.23±4.31
PBS+AA组	5	91.5±31.6^a^	0.39±0.81^a^	90.85±121.90^a^	0.38±0.38^a^
CLO+AA组	5	110.6±24.1^b^	1.32±2.45^a^	461.13±483.45^b^	1.10±2.31

注：对照组：正常F1小鼠；PBS+AA组：隔日1次200 µl PBS脂质体尾静脉注射（共4次）的F1再生障碍性贫血模型小鼠；CLO+AA组：隔日1次200 µl Clodronate脂质体尾静脉注射（共4次）的F1再生障碍性贫血模型小鼠。与对照组相比，^a^*P*<0.05；与PBS+AA组相比，^b^*P*<0.05

**图1:**
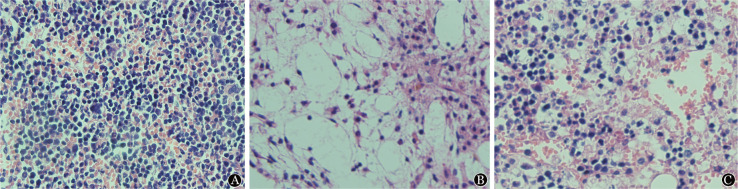
各处理组小鼠骨髓病理结果（×100，HE染色） A：对照组（正常F1小鼠）；B：PBS+AA组（隔日1次共4次200 µl PBS脂质体尾静脉注射的F1再生障碍性贫血模型小鼠）；C：CLO+AA组（隔日1次共4次200 µl Clodronate脂质体尾静脉注射的F1再生障碍性贫血模型小鼠）

2. 小鼠骨髓CD4^+^/CD8^+^ T淋巴细胞亚群比较：见表2。与对照组小鼠相比，PBS+AA组小鼠的CD4^+^和CD8^+^ T淋巴细胞比值均明显升高（*P*值均<0.05）。去除巨噬细胞后，相较PBS+AA组，CLO+AA组小鼠CD4^+^和CD8^+^ T淋巴细胞比例均显著降低（*P*值均< 0.05），且CD8^+^ T细胞减少的程度更高。

**表2:** 各处理组小鼠骨髓淋巴细胞亚群的比较（％，*x±s*）

组别	鼠数	CD4^+^细胞比例	CD8^+^细胞比例
对照组	3	4.62±0.75	4.98±0.17
PBS+AA组	5	18.50±10.17^a^	36.23±6.40^a^
CLO+AA组	5	7.58±8.00^ab^	6.67±5.78^ab^

注：对照组：正常F1小鼠；PBS+AA组：隔日1次200 µl PBS脂质体尾静脉注射（共4次）的F1再生障碍性贫血模型小鼠；CLO+AA组：隔日1次200 µl Clodronate脂质体尾静脉注射（共4次）的F1再生障碍性贫血模型小鼠。与对照组相比，^a^*P*<0.05；与PBS+AA组相比，^b^*P*<0.05

3. 小鼠外周血血浆IFN-γ、TNF-α、GM-CSF、G-CSF、EPO和TPO水平的比较：见表3。与对照组小鼠相比，PBS+AA组小鼠外周血血浆中IFN-γ、TNF-α、GM-CSF、G-CSF、EPO和TPO的水平均显著增高（*P*值均<0.05）；与PBS+AA组相比，CLO+AA组小鼠外周血血浆中IFN-γ、TNF-α水平明显降低；正性造血调控因子GM-CSF、G-CSF、EPO和TPO水平也有所下降，但仍然显著高于对照组，差异均有统计学意义（*P*值均<0.05）。

**表3:** 各处理组小鼠外周血血浆细胞因子水平的比较（g/L，*x±s*）

组别	鼠数	IFN-γ	TNF-α	GM-CSF	G-CSF	EPO	TPO
对照组	3	110.21±17.32	12.19±1.56	3.07±0.48	141.71±39.95	155.45±46.61	2.02±1.22
PBS+AA组	5	602.37±104.62^a^	34.46±1.42^a^	64.85±12.25^a^	17 784.16±488.36^a^	9 667.31±4 501.95^a^	11.67±2.86^a^
CLO+AA组	5	303.01±87.22^ab^	23.25±4.21^ab^	9.32±2.00^ab^	5 891.78±2 632.39^ab^	2 078.02±897.56^ab^	6.36±2.09^ab^

注：对照组：正常F1小鼠；PBS+AA组：隔日1次200 µl PBS脂质体尾静脉注射（共4次）的F1再生障碍性贫血模型小鼠；CLO+AA组：隔日1次200 µl Clodronate脂质体尾静脉注射（共4次）的F1再生障碍性贫血模型小鼠。与对照组相比，^a^*P*<0.05；与PBS+AA组相比，^b^*P*<0.05

4. 小鼠脾脏总巨噬细胞及M1/M2亚群的比较：与PBS组相比，CLO组小鼠的脾脏总巨噬细胞比例明显下降，差异有统计学意义（*P*<0.05），提示脾脏中巨噬细胞的清除效果较为显著；且CLO组的M1型巨噬细胞比例显著降低，差异有统计学意义（*P*<0.05），M2型巨噬细胞比例差异无统计学意义。造模后，与PBS+AA组相比，CLO+AA组脾脏总巨噬细胞及M1巨噬细胞亚型均显著下降（*P*值均<0.01），降低程度与造模前差异均无统计学意义（表4）。

**表4:** 各处理组小鼠脾脏总巨噬细胞及MI/M2亚群比例的比较（％，*x±s*）

组别	鼠数	总巨噬细胞	M1型	M2型
对照组	3	2.71±0.44	0.49±0.14	0.38±0.03
PBS组	4	4.16±0.68	1.11±0.37	0.42±0.16
CLO组	4	2.17±0.54^b^	0.13±0.07^b^	0.94±0.52
PBS+AA组	5	7.08±2.81	2.32±0.75	1.89±1.06
CLO+AA组	5	4.19±1.11^c^	0.96±0.41^c^	1.42±0.47

注：对照组：正常F1小鼠；PBS组：隔日1次200 µl PBS脂质体尾静脉注射（共4次）的正常F1小鼠；CLO组：隔日1次200 µl Clodronate脂质体尾静脉注射（共4次）的正常F1小鼠；PBS+AA组：隔日1次200 µl PBS脂质体尾静脉注射（共4次）的F1再生障碍性贫血模型小鼠；CLO+AA组：隔日1次200 µl Clodronate脂质体尾静脉注射（共4次）的F1再生障碍性贫血模型小鼠。与对照组相比，^a^*P*<0.05；与PBS组相比，^b^*P*<0.05；与PBS+AA组相比，^c^*P*<0.05

5. 小鼠骨髓总巨噬细胞及M1/M2亚群的比较：与PBS组相比，CLO组小鼠的骨髓总巨噬细胞比例明显下降，差异有统计学意义（*P*<0.05），提示骨髓中巨噬细胞的清除效果同样较为显著，CLO组的M1型巨噬细胞比例显著降低，差异有统计学意义（*P*<0.05）。造模后，与PBS+AA组相比，CLO+AA组骨髓总巨噬细胞及M1巨噬细胞亚型均显著下降（*P*值均<0.05），降低程度与造模前差异无统计学意义（表5）。

**表5:** 各组小鼠骨髓总巨噬细胞及MI/M2亚群比例的比较（％，*x±s*）

组别	鼠数	总巨噬细胞	M1型	M2型
对照组	3	30.13±2.68	2.29±0.48	0.50±0.15
PBS组	4	28.15±0.87	4.07±1.74	0.75±0.37
CLO组	4	11.37±1.78^ab^	1.62±0.45^b^	1.73±0.92
PBS+AA组	5	18.82±10.91	3.25±0.46	5.65±6.09
CLO+AA组	5	6.68±10.68^c^	0.77±0.46^c^	0.82±1.34

注：对照组：正常F1小鼠；PBS组：隔日1次200 µl PBS脂质体尾静脉注射（共4次）的正常F1小鼠；CLO组：隔日1次200 µl Clodronate脂质体尾静脉注射（共4次）的正常F1小鼠；PBS+AA组：隔日1次200 µl PBS脂质体尾静脉注射（共4次）的F1再生障碍性贫血模型小鼠；CLO+AA组：隔日1次200 µl Clodronate脂质体尾静脉注射（共4次）的F1再生障碍性贫血模型小鼠。与对照组相比，^a^*P*<0.05；与PBS组相比，^b^*P*<0.05；与PBS+AA组相比，^c^*P*<0.05

## 讨论

本实验采用本课题组改良研制的新型F1 AA小鼠模型，相较于Sun等[6]的动物模型不同之处主要包括：（1）受鼠品种不同：虽然供鼠均为C57BL/6小鼠，但本实验的受鼠为 B6D2F1杂交鼠，Sun等[6]为CByB6F1杂交鼠；（2）辐照源不同：本实验采用X射线对受鼠进行5 Gy的全身辐照，Sun等[6]采用^137^γ射线进行5 Gy的全身辐照；（3）输注淋巴结的数量及途径不同：本实验每只受鼠腹腔注射约8×10^6^个淋巴结细胞，Sun等[6]每只受鼠尾静脉注射约5×10^6^个淋巴结细胞。在不同小鼠模型中重新验证巨噬细胞对AA发病的影响，更能证实巨噬细胞的病理作用。并有利于进一步深入研究。

巨噬细胞根据活化状态和发挥功能的不同，可分为M1型（即经典活化的巨噬细胞）和M2型（即替代活化的巨噬细胞）[8]。巨噬细胞作为一种极具异质性的细胞群体，在复杂的微环境中，会表现出独特的表型和功能[9]。Mantovani等[10]认为巨噬细胞存在一系列连续的功能状态，而M1型和M2型巨噬细胞是这一连续状态两个极端。M1型巨噬细胞分泌促炎性的细胞因子和趋化因子，并专职提呈抗原，参与正向免疫应答，发挥免疫监视的功能；M2型巨噬细胞仅有较弱抗原提呈能力，并通过分泌抑制性的细胞因子如IL-10和（或）TGF-β等下调免疫应答，在免疫调节中发挥重要作用[10]–[11]。因为M1型与M2型巨噬细胞对免疫的调控作用正好相反，巨噬细胞对造血也具有双向调控作用。一方面巨噬细胞在造血干细胞移植后的小鼠中通过促进基质细胞的功能，减轻骨髓的炎性损伤，促进造血重建，发挥其促进造血的作用[12]。另一方面在骨髓衰竭小鼠模型中，宿主小鼠中的巨噬细胞是介导淋巴细胞攻击骨髓细胞必不可少的成分[6]，且临床上移植患者骨髓中巨噬细胞、CD8^+^ T 细胞的增加预示异基因造血干细胞移植或脐血移植的失败，则证实了巨噬细胞抑制造血的作用[13]。因此，通过表型鉴定巨噬细胞的类型，对研究巨噬细胞在不同生理和病理条件下所发挥的功能具有重要意义[14]。

巨噬细胞源自单核细胞，如何参与AA的发病目前尚不清楚。已知巨噬细胞的活化方式主要有以下三种：①由IFN-γ或细菌脂多糖（LPS）诱导产生，激活Th1型细胞，分泌多种炎症因子（如IL-1、IL-6、IL-12、IL-23、TNF 等）及趋化因子（如CCL1、CCL2、CCL3、CCL4、CCL5 等）；②由IL-4、IL-10、IL-13、糖皮质激素等诱导，抑制免疫应答，参与组织修复；③由诱导巨噬细胞经典活化的刺激分子在IgG免疫复合物存在的情况下诱导产生，终止IL-12合成并产生大量的IL-10，参与Th2型细胞的免疫应答[15]–[17]。

既往的研究已经表明，AA的发生发展与T细胞（尤其是CD8^+^ T淋巴细胞）功能亢进引起的造血组织损伤关系密切[18]–[19]。激活的T淋巴细胞在骨髓中迅速克隆增殖并大量释放Ⅰ型淋巴因子IFN-γ、TNF-α等炎症因子导致骨髓造血干细胞（HSC）凋亡。我们的实验发现，提前去除巨噬细胞的AA小鼠骨髓中CD8^+^ T淋巴细胞的浸润明显减少，外周血循环中的TNF-α和IFN-γ浓度也相应降低，与文献[18]–[19]的研究结果一致。

长期以来，IFN-γ和TNF-α一直被认为是破坏骨髓造血细胞的关键细胞因子[1],[20]–[21]。IFN-γ作为巨噬细胞、自然杀伤细胞等的主要激活剂，在先天免疫和后天免疫中都发挥着重要作用，同时也是导致AA发病的重要成分。体外实验证实IFN-γ可以直接抑制人HSC的增殖，而免疫介导的骨髓衰竭也与IFN-γ在T细胞中过表达相关[22]–[23]。AA初诊患者的IFN-γ信号通路存在基因的异常，比如T-bet基因的上调[24]。另外，IFN-γ还可以刺激骨髓造血干/祖细胞（HSPC）表面Fas的表达，促进其与活化的T细胞结合进入Fas/FasL凋亡途径[25]。TNF-α也是重要的造血调节因子且与分泌水平密切相关，低水平TNF-α可直接或者与IL-3、GM-CSF等造血因子协同促进人HSPC的增殖，而高分泌水平的TNF-α则会抑制造血[26]。大量的临床资料证实，AA 患者TNF-α水平与正常人相比明显增高，而且TNF-α水平与外周血细胞数量呈负相关[27]–[29]。近年研究认为凋亡是AA的一个主要的病理过程，而TNF-α/TNFR1介导细胞凋亡的途径已被人们所熟知，即TNF-α与TNFR1结合后，通过激活下游的caspase-8及各种效应分子，产生级联激活反应，最终引起靶细胞凋亡。有文献证实，AA患者T细胞中的TNF-α和骨髓CD34^+^细胞上的TNF-α受体（TNF-αR）均上调[30]–[31]，然而也有研究显示，TNF-α可能主要通过Fas/FasL途径介导骨髓HSC的凋亡[32]。Sun等[6]则认为TNF-α通过刺激T淋巴细胞分泌大量IFN-γ，加速骨髓衰竭。

此外，本实验我们发现，造模后的PBS+AA组小鼠外周血GM-CSF、G-CSF、EPO、TPO的水平均明显高于对照组，这与大多数AA患者的临床表现一致[33]。去除巨噬细胞后，CLO+AA组小鼠外周WBC、HGB、PLT高于PBS+AA组，骨髓造血情况也好于PBS+AA组，但是GM-CSF、G-CSF和TPO 的水平仍然显著高于正常对照。确实有文献报道，AA患者无论缓解与否，其TPO水平都显著高于健康对照组[34]。这提示，AA小鼠骨髓中HSC衰竭可能不是由于内源性GM-CSF、G-CSF和TPO分泌不足引起的，可能由于HSC受损，对其敏感性降低[35]；或靶细胞及其受体受损，使其失去正向调控作用等[36]–[37]。

流式检测结果显示，注射Clodronate脂质体后，小鼠骨髓及脾脏中总巨噬细胞的比例均明显减少且减少程度相当。均以M1型巨噬细胞减少为主，M2型巨噬细胞的比例反而有增加趋势。对此，根据Sun等[6]的研究结果，我们作如下推测：PBS+AA组小鼠由于AA，骨髓微环境中存在T细胞分泌的大量IFN-γ，可诱导M0向M1分化[38]–[39]，所以骨髓中M1巨噬细胞占比较多。巨噬细胞，尤其是M1型，是TNF-α的主要来源。CLO+AA组小鼠由于大量耗竭了M1巨噬细胞，使得骨髓微环境中TNF-α产生显著减少，对细胞毒性T细胞的刺激降低，其分泌的IFN-γ相应减少[6]，形成良性循环，小鼠骨髓衰竭得以缓解。

综上所述，机体内M1/M2巨噬细胞亚群的变化及造血调控因子分泌的紊乱，可能会促进骨髓衰竭的形成。且与M2型巨噬细胞相比，M1型巨噬细胞在AA的发病中起到更加关键的作用。当然，骨髓微环境是一个相当复杂的动态平衡系统，更多的、更精确的巨噬细胞分型以及巨噬细胞与造血正负调控因子之间相互作用的具体机制还有待进一步研究，而且在AA患者骨髓中巨噬细胞所起的作用是否与小鼠模型中完全相同也有待于进一步探索。
